# The prevalence of hypertension and abnormal kidney function in children with sickle cell disease –a cross sectional review

**DOI:** 10.1186/1471-2369-14-237

**Published:** 2013-10-30

**Authors:** Prasad Bodas, Alex Huang, Mary Ann O’Riordan, John R Sedor, Katherine MacRae Dell

**Affiliations:** 1 Department of Pediatrics, Division of Hematology and Oncology, Akron Children’s Hospital, Akron, OH 44302, USA; 2 Department of Pediatrics, Case Western Reserve University, Cleveland, OH 44106, USA; 3 Department of Pediatrics, Rainbow Babies and Children’s Hospital, Cleveland, OH 44106, USA; 4 CWRU Center for the Study of Kidney Disease and Biology, Case Western Reserve University, Cleveland, OH 44106, USA

**Keywords:** Sickle cell disease, Nephropathy, Chronic kidney disease, Hypertension

## Abstract

**Background:**

Renal disease is a known contributor to mortality in adults with sickle cell disease (SCD) and renal abnormalities are evident in childhood. Hyperfiltration (evidenced by elevated glomerular filtration rate, GFR) occurs in children with SCD early in disease. However, the incidence of low GFR (<90 ml/min/1.73 m2) suggestive of chronic kidney disease (CKD), is not well established. The prevalence of hypertension is also not well known. The goal of this study was to determine the prevalence of hypertension and CKD in a cohort of children with SCD.

**Methods:**

We performed a retrospective chart review of patients followed at the Rainbow Babies and Children’s Sickle Cell Disease Clinic who were seen during routine follow up visits. Inclusion criteria were all patients ages 3–18. Exclusion criteria included recent (within 2 weeks) hospitalization and/or episode of acute chest, pain crises, febrile illness or red blood cell transfusion. Data collected included serum creatinine, blood pressure and history of sickle cell complications (acute chest syndrome, stroke or stroke risk). Estimated GFR (eGFR) was calculated using the updated Schwartz creatinine-based estimating formula. Analysis examined the associations among eGFR, blood pressure and sickle cell complications. The Institutional Review Board at University Hospitals Case Medical Center approved this study.

**Results:**

A total of 48 children had complete data available. Mean eGFR was 140 mL/min/1.73 m^2^ +/- 34.9 (range 71.9-404.2 mL/min/1.73 m^2^). Four patients (8.3%) had eGFRs < 90 mL/min/1.73 m^2^, 35 patients (72.9%) had eGFRs > 120 mL/min/1.73 m^2^ and 9 patients (18.8%) had eGFRs in the normal range. Eight patients (16.7%) had evidence of elevated blood pressures (pre-hypertension or hypertension). There was no correlation between eGFR and age, and no association of eGFR with acute chest or stroke risk.

**Conclusion:**

In this SCD cohort, we identified abnormally low eGFR (suggestive of early CKD) in 8.3% of patients and elevated blood pressure in 16.7%. These findings are in contrast to other published studies that show primarily normal or elevated GFR and the absence of or minimal hypertension. These findings indicate that elevated blood pressure and decreased eGFR are not uncommon in children with SCD, and should be more rigorously studied.

## Background

Chronic kidney disease (CKD) in patients with sickle cell disease (SCD) is a risk factor for early death. This mortality association is stronger than that of an episode of the acute chest syndrome, febrile episode with positive blood culture, acute stroke, right upper quadrant syndrome, or severe acute anemia [1]. Retrospective studies suggest that nephropathy of varying severity occurs in 5-18% of sickle cell patients, depending on the age studied [2-5], and up to one third of adults with SCD will develop CKD [1].

Renal consequences of SCD are evident in early life, and can involve multiple segments of the nephron. Impaired urine concentrating ability is almost universally present early in childhood. Hematuria and overt papillary necrosis can develop as a result of medullary ischemia [6-10]. Glomerular filtration rate (GFR) is elevated. Increased renal blood flow and subsequent glomerular hypertrophy are thought to contribute to the development of proteinuria and progressive glomerulosclerosis (sickle cell glomerulopathy). Renal complications may not become clinically overt until the second decade of life. However, up to 20% of young adults with SCD exhibit nephrotic range (heavy) proteinuria [11-13].

Because the survival rate of SCD patients is increasing, CKD will likely play a greater role in morbidity and mortality in the future. Characterizing the early kidney disease phenotype will be important in designing clinical trials. The goal of this study is to describe the prevalence of hypertension and abnormal renal function in a well-characterized group of children with SCD.

## Methods

This study was approved by the Institutional Review Board at University Hospitals Case Medical Center.

We retrospectively reviewed records of children ages 3–18 years actively receiving care at the University Hospitals- Rainbow Babies and Children’s Pediatric Sickle Cell Clinic between March 2010- January 2011. Data obtained from the most recent well outpatient visit included age, gender, height, blood pressure, and serum creatinine concentration. Blood pressure measurements reflect a single seated oscillometric measurement obtained during clinic vital sign assessments using a Dynamap® device. The serum creatinine was the most recent one obtained during a routine outpatient visit. Because serum creatinine can be affected by hydration status and/or medication use (e.g. non-steroidal anti-inflammatory agents) in the context of an acute illness, the value utilized was one that was obtained at least 2 weeks after an acute illness.

Sickle cell genotype was identified from the patient record, determined by hemoglobin electrophoresis or high-performance liquid chromatography (HPLC). We also collected historical information regarding the occurrence of two severe sickle cell complications: (1) acute chest syndrome (ACS); and (2) stroke or high risk for stroke as identified by trans-cranial Doppler screening [14]. Patients who had received a blood transfusion or had known illness (including any sickle cell crisis) within two weeks prior to the visit were excluded, as were those for whom complete data were not available.

Renal function was assessed by determination of eGFRs calculated using the updated “bedside” Schwartz equation:

$$\begin{array}{l} \mathrm{GFR}\left(\mathrm{mL}/\min/1.{73m}^{2}\right)=\left[0.413\times \text{Height}\left(\mathrm{cm}\right)\right]\\ \;/\text{Serum}\;\text{creatinine}\left(\mathrm{mg}/\mathrm{dL}\right) \end{array}$$

This formula, which utilizes standardized serum Isotope Dilution Mass Spectrophotometry (IDMS) traceable creatinine measurements has been validated in pediatric patients with and without CKD using measured glomerular filtration methodologies (iohexol clearance) [15-18]. All serum creatinine values used in this study were obtained using an IDMS-traceable serum creatinine assay. For blood pressure analysis, pre-hypertension (systolic BP in 90- <95th% for age, height, and gender) and hypertension (systolic BP ≥95th% for age, height, and gender) were defined based on published normative data [19]. High stroke risk was defined as the history of a stroke and/or abnormal trans-cranial Doppler velocity screening.

### Statistical analysis

Pearson correlation coefficients were used to determine the correlation between eGFR and age and systolic and diastolic blood pressure percentiles. The association between eGFR and the presence of clinical events including ACS, high stroke risk based on trans-cranial Doppler velocity screening, and genotype was determined using distributionally appropriate two-sample tests (t tests were used for normally distributed data and Wilcoxon rank sum tests were used for non-normally distributed data).

## Results

Data from 48 patients were included in this analysis. Demographics, clinical characteristics, and measurements are shown in Table 1. The majority of subjects (32/48) had HbSS disease, with the remainder having HbS/beta0-thalassemia (1/48), HbS/C (9/48), or HbS/beta + thalassemia (6/48) genotype. Mean age was 12+/- 3.1 years (range 3–17 years) and 52% of subjects were male. A history of at least one episode of the acute chest syndrome was present in 19/48 (39.6%) patients. None of the patients had stroke, but 6/48 (12.5%) had high stroke risk noted by trans-cranial Doppler screening.

**Table 1:** Demographic and clinical characteristics of study population

**n**	**48**
Male: Female	25: 23
Age, mean (range in years)	12+/-3.1 (3–17)
History of acute chest syndrome, n (%)	19 (39.6)
Elevated stroke risk, n (%)	6 (12.5)
Pre-hypertension, n (%)	4 (8.3)
Hypertension, n (%)	4 (8.3)
eGFR* mean (range)	140+/-34.9 (71.9–404.2)
eGFR* sugroups	
<90	4
90-120	9
>120	35
**Sickle cell genotype**	
SS, n (%)	32 (66.7)
SC, n (%)	9 (18.8)
S beta + -thalassemia, n (%)	6 (12.5)
S beta 0-thalassemia, n (%)	1 (2.1)

* eGFR expressed as ml/min/1.73 m^2^.

Mean eGFR was 140 mL/min/1.73 m^2^ +/- 34.9 (range 71.9- 404.2). Four patients (8.3%) had eGFR below the normal range (<90 mL/min/1.73 m2), suggestive of CKD. Conversely, 35 patients (72.9%) had eGFR above the normal range (>120 mL/min/1.73 m2). A total of 8 patients had elevated blood pressures. Pre-hypertension was present in 4/48 (8.3%) and overt hypertension was present in 4/48 (8.3%) of patients. The characteristics of the patients in the low eGFR, normal or elevated eGFR, elevated BP, and non-elevated BP are summarized in Table 2.

**Table 2:** Characteristics of patients by eGFR and blood pressure

	**Low GFR (eGFR <90 ml/min/1.72 m**^**2**^**)**	**Normal or elevated eGFR**	**Elevated BP (hypertension or pre- hypertension)**	**Non-elevated BP**
n (%)	4/ 48 (8.3%)	44/ 48 (91.7%)	8/ 48 (16.6%)	40/48 (83.3%)
Male: Female	2:2	23:21	3:5	22:18
Age, mean (range in years)	12.5 +/- 2.1 (10–15)	12 +/- 3.2 (3–17)	10.5 +/- 2.4 (6–13)	12.3 +/- 3.2 (3–17)
**Genotype, n (% of total patients)**				
SC	2 (4.2)	30 (62.5)	6 (12.5)	26 (54.2)
SS	1 (2.1)	8 (16.6)	1 (2.1)	8 (16.7)
S beta + -thalassemia	1 (2.1)	5 (10.4)	1 (2.1)	5 (10.4)
S beta 0-thalassemia	0 (0)	1 (2.1)	0 (0)	1 (2.1)

There was no significant correlation between eGFR and age or blood pressure. eGFR was not associated with gender, genotype, or the presence or absence of acute chest history or stroke risk.

## Discussion

The results of this study demonstrate the presence of abnormal kidney function (eGFR > 120 or <90 ml/min/1.73 m2) in a significant proportion of the study population. The majority of these (35/48) had elevated eGFR consistent with hyperfiltration. This finding is consistent with previous published reports in SCD. In contrast, low eGFR values (< 90 mL/min/1.73 m^2^) were noted in 8.3% of our population, a cohort with a mean age of 12.5 years. This finding is contrary to some published reports which have suggested CKD is a later finding [2,3]. Of note, a more recent cross-sectional study by McPherson Yee, et al. [20] reported an 11.6% prevalence of eGFR <90 mil/min/1.73 m2 in their patient population (mean age 11.4 +/- 4.5 years), supporting our finding that CKD may be more common in the pediatric population than previously recognized. In contrast to that study and other previously published reports [2-4], however, we found no correlation between age and eGFR. The differences between our findings and those of prior studies may be due to the relatively small sample size of our study, or the age distribution of our cohort, which had no adults and only one child over age 16 years.

Interestingly, in our analysis we were unable to show correlation of eGFR and episodes of the acute chest syndrome or high stroke risk. This could suggest that the factors contributing to the development of these complications may be different than those influencing the development of CKD. Alternatively, the absence of a correlation could have been related to the relatively small sample size and few clinical events. One factor that could uniquely contribute to the development of CKD is the presence of one or more APOL1 “risk variants,” which has been shown to contribute to the development of non-diabetic CKD [21,22]. APOL1 genotyping was beyond the scope of this study but would be an important area of future investigation.

Taken together, our data and that of others suggest that abnormally low GFR is not an uncommon problem for children with SCD, and renal function should be assessed routinely in these patients. However, determining the optimal method for assessing renal function in the clinical setting as well as frequency of monitoring can be problematic. Quantification of inulin clearance in a timed urine collection is considered the “gold standard” measurement of GFR. However, this method is technically demanding, requiring bladder catheterization and 24 hour urine collection [23,24]. Nuclear medicine based techniques such as iohexol or technetium ^99^ m– labeled diethylenetriaminepentaacetic acid (DTPA) provide accurate results comparing favorably with inulin clearance and does not require timed urine collections [25-28]. However these procedures may not be available at all centers. Because these techniques are costly and time consuming, their feasibility for clinic-based screening of renal function is limited. Creatinine-based estimating formulas (such as the bedside Schwartz formula in children or the MDRD formula for adults) or 24 hour urine collections for creatinine clearance are less expensive and relatively easy to obtain but also have limitations. Importantly, increased creatinine secretion due to hyperfiltration or tubular dysfunction can result in overestimation of GFR.

Pediatric patients with SCD have typically been thought to have low normal blood pressures. In a study of 85 children, Aygun et al. [29] identified no hypertensive patients despite abnormal calculated and measured GFRs. In addition, in a Saudi Arabian cohort of 69 children with SCD aged 1–16 years old, blood pressure measurements were within normal range [30]. However, in the present study, 16.6% of patients had elevated blood pressures (overt hypertension in 8.3% and prehypertension in 8.3%). Interestingly, the prevalence of pre-hypertension and hypertension in our cohort is not statistically different than what has been found in school based screenings of African American children in this country [31]. Of note, investigators in a recent study demonstrated a 10.3% prevalence of hypertension in a cohort of thirty-eight children with SCD, based on in-clinic blood pressure screening [32]. Importantly, that study utilized ambulatory blood pressure monitoring (ABPM, the gold standard for diagnosing hypertension) to determine the true prevalence of hypertension and found 43.6% had ambulatory hypertension. This suggests that the incidence of “masked” hypertension may be quite high in pediatric SCD patients, with rates approaching those of children with clear evidence of CKD [33]. These findings coupled with our data suggest that hypertension may be under diagnosed in children with SCD when using standard clinic based assessments. This highlights the need for identification and evaluation of elevated blood pressures in the clinic setting using standardized, validated methods.

Although rates of hypertension are high in patients with CKD, we were unable to demonstrate a correlation between eGFR and systolic or diastolic hypertension in our population. This lack of correlation may have been due to the relatively small number of patients with CKD. However, it does suggest that hypertension is present in this population in the absence of clear evidence of renal dysfunction.

Our study had several important limitations. These included a relatively small sample size, and the retrospective design. Utilization of data obtained during routine clinical practice and abstracted from retrospective chart review imposed significant constraints. Blood pressure measurements recorded at each visit were collected according to the standard clinical practice, and not in accordance with approved methodology [19]. Because repeated measures were not performed and/or hypertension was not confirmed with manual readings, it is possible that the prevalence of true hypertension may be lower than our data suggest. In addition, as discussed above, creatinine-based eGFRs, which were utilized in the present study, have important limitations.

One important additional limitation is the absence of data on albumin excretion. Albuminuria has been shown to be an early marker for pathology in various kidney diseases. Other investigators have demonstrated that albuminuria may be an important early marker of kidney disease in children with SCD, with Yee, et al. demonstrating prevalence rates of 20.7% [20]. In that study, albuminuria correlated with older age and lower hemoglobin concentration but not with low eGFR. Imuetinyan et al. reported an albuminuria prevalence of 20.3% [30]. Among 90 children with SCD, Becton et al. found albuminuria to be present in 15.5% [34]. However, it should be noted that albuminuria may regress in some instances. In diabetes, one of the best studied causes of CKD, albuminuria may regress in up to 25% of patients [35]. It is not known whether this occurs in SCD and the role of albuminuria in predicting progression to end stage renal disease (ESRD) in this patient population remains a valuable area of study.

In addition to albuminuria, multiple investigators are pursuing novel candidate biomarkers that are not currently used in common clinical practice. Urinary levels of renal kallikrein, [36,37] transforming growth factor beta-1 [38], endothelin-1 [39], and KIM-1 and NAG [40] have been, or are currently being investigated, and show promise.

## Conclusions

In conclusion, the results of this retrospective study identified abnormally low eGFR (suggestive of early CKD) in 8% of patients and elevated blood pressure in 16.6%. These findings highlight the importance of performing standardized blood pressure measurements and monitoring serum creatinine and urine albumin excretion on a regular basis in this population. The subset of children with SCD who have low eGFR, hypertension and/or albuminuria may be at particular risk for development of overt sickle cell nephropathy and/or advanced CKD and merit close attention. Our findings highlight the need for a larger prospective longitudinal study aimed at identifying and following children at risk for CKD to determine which factors are predictive of progression. In addition, newer markers of early sickle cell nephropathy (such as novel urine and serum biomarkers, or quantitative MRI) [41] should be explored. A clinical measure that identifies patients in the early stages of sickle cell nephropathy would permit targeted monitoring and treatment to high-risk patients and facilitate clinical trials of new therapies.

## Abbreviations

SCD: Sickle cell disease; GFR: Glomerular filtration rate1; CKD: Chronic kidney disease; eGFR: Estimated glomerular filtration rate; HPLC: High performance liquid chromatography; ACS: Acute chest syndrome; MRI: Magnetic resonance imaging; ABPM: Ambulatory blood pressure monitoring; ESRD: End stage renal disease.

## Competing interests

The authors have no competing interests to disclose.

## Authors’ contributions

PB conceived of the study, participated in its design, performed the background review and performed the cross sectional analysis, and drafted the manuscript. AH participated in manuscript review and design. MAO performed the statistical analysis. JRS helped draft the manuscript. KD conceived of the study, participated in its design, and helped to draft the manuscript. All authors read the final manuscript.

## Pre-publication history

The pre-publication history for this paper can be accessed here:

http://www.biomedcentral.com/1471-2369/14/237/prepub
